# Upconversion Nanoparticles Decorated with Polysialic Acid for Solid Tumors Visualization In Vivo

**DOI:** 10.1134/S1607672921020034

**Published:** 2021-03-05

**Authors:** P. A. Demina, N. V. Sholina, R. A. Akasov, D. A. Khochenkov, A. V. Nechaev, I. V. Balalaeva, E. V. Khaydukov, A. N. Generalova, S. M. Deev

**Affiliations:** 1 Shemyakin–Ovchinnikov Institute of Bioorganic Chemistry, Russian Academy of Sciences, Moscow, Russia; 2 Sechenov First Moscow State Medical University, Moscow, Russia; 3 Federal Research Center “Crystallography and Photonics,” Russian Academy of Sciences, Moscow, Russia; 4 Blokhin National Medical Research Center for Oncology, Ministry of Health of the Russian Federation, Moscow, Russia; 5 Lomonosov Moscow State University of Fine Chemical Technologies, Moscow, Russia; 6 Lobachevsky State University of Nizhny Novgorod, Nizhny Novgorod, Russia

**Keywords:** upconversion nanophosphors, bioimaging, surface functionalization, polysialic acid

## Abstract

Upconversion nanoparticles (UCNPs) are a promising nanoplatform for bioreagent formation for in vivo imaging, which emit UV and blue light under the action of near-infrared radiation, providing deep tissue penetration and maintaining a high signal-to-noise ratio. In the case of solid tumor visualization, the UCNP surface functionalization is required to ensure a long circulation time, biocompatibility, and non-toxicity. The effective UCNP accumulation in the solid tumors is determined by the disturbed architecture of the vascular network and lymphatic drainage. This work demonstrates an approach to the UCNP biofunctionalization with endogenous polysialic acid for in vivo bioreagent formation. Bioreagents possess a low level of nonspecific protein adsorption and macrophage uptake, which allow the prolongation of the circulation time in the bloodstream up to 3 h. This leads to an intense photoluminescent signal in the tumor.

## INTRODUCTION

Currently, the main trends in the development of medicine are associated with the development of a personalized approach to diagnostics and therapy. Methods based on the use of nanoparticles serving as a nanoplatform for creating multimodal reagents have significant prospects for solving these problems. As such nanoparticles, inorganic upconversion nanoparticles (UCNPs) that are activated with near-IR light, which allows solid tumor imaging, are of great interest [1]. In this case, the biofunctionalization of the UCNPs surface is responsible for the bioanalysis format, for example, in the form of passive, targeted, or magnetically controlled (in the case of functionalization with magnetic nanoparticles) delivery [2]. For the efficient accumulation of UCNPs in solid tumors, it is necessary to solve the problem of their long-term circulation in the bloodstream, which is associated with the creation of a biocompatible non-toxic surface that prevents the aggregation of nanoparticles (in particular, the aggregation through the formation of complexes with blood proteins) and does not induce an immune response. In this case, UCNPs can be delivered to the tumor via a passive mechanism based on the effect of increased permeability and retention of nanoparticles in the vessels (EPR effect), which is associated with hypervascularization and disturbance of lymphatic drainage in the tumor [3]. Such biofunctionalization of the UCNP surface can be realized using an endogenous polymer. In this work, we used polysialic acid (PSA) as such a polymer. PSA is a highly hydrophilic non-immunogenic and non-toxic homopolymer of α-2,8 5-*N*-acetylneuraminic acid, which is degraded by neuraminidase to biocompatible sialic acid [4]. In an aquatic environment, PSA exists in the form of associates with water molecules, which significantly reduces the adsorption of blood proteins and, as a result, increases the circulation time of nanoparticles in the bloodstream [5]. For example, obtaining conjugates of PSA with proteins made it possible to obtain drugs with improved pharmacokinetics, which was demonstrated in [6].

The aim of this study was to develop a method for biofunctionalization of UCNPs using PSA, which allows the creation of photoluminescent bioreagents for solid tumor imaging in vivo.

## RESULTS AND DISCUSSION

**Fig. 1.**
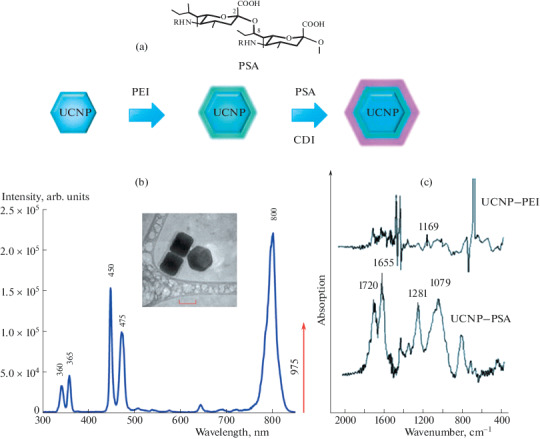
(a) The structural formula of polysialic acid and the scheme for the modification of UCNPs with PSA. (b) Photoluminescence spectrum of UCNPs at an excitation wavelength of 975 nm and UCNPs, scale 50 nm. (c) IR-Fourier spectra after modification UCNP–PEI (top) and UCNP–PSA (bottom).

UCNPs with the core/shell structure (NaYF_4_: Yb^3+^/Tm^3+^@NaYF_4_) with an average size of 90 ± 6 nm were synthesized using the solvothermal method described earlier [7]. The hydrophobic surface of UCNPs was modified with PSA in two stages. First, the surface was hydrophilized with polyethyleneimine (PEI) using the solvent exchange method. For this purpose, UCNP dispersed in chloroform was mixed with a PEI solution in the same solvent. The mixture was incubated for 1 h under stirring to allow the polymer to adsorb on the surface, after which it was added dropwise to water under sonication. After evaporation of the solvent, an aqueous dispersion of nanoparticles modified with PEI with amino groups exposed on the surface, having a zeta potential of +42 mV, was obtained [8]. At the second stage, UCNPs were modified with polysialic acid due to the formation of a covalent bond between the amino group of PEI and the carboxyl group of the acid using the carbodiimide activation procedure (Fig. 1a). As a result of the modification, the UCNP–PSA nanocomplexes (zeta potential, –32 mV), colloidly stable in buffer solutions, were obtained, which retained their aggregate stability for 2 months. The presence of PSA on the UCNP surface was confirmed by FTIR spectroscopy by the appearance of the main characteristic PSA peaks in the region of 1079, 1281, 1655, and 1720 cm^–1^ (Fig. 1c).

**Fig. 2.**
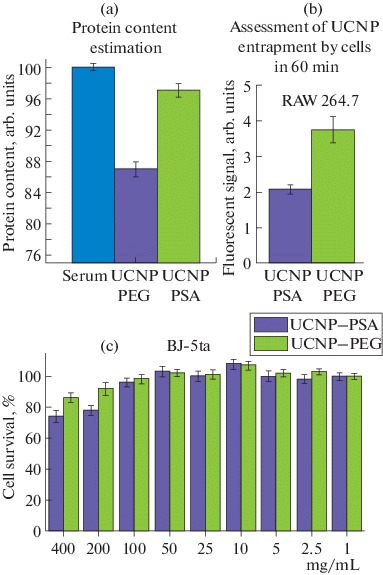
(a) The protein content in the supernatant after the incubation of the UCNP samples in the serum: UCNP–PEG, UCNP–PSA, and control (pure serum). (b) Normalized fluorescent signal of UCNP–PSA and UCNP–PEG accumulated in mouse macrophages RAW 264.7. (c) Survival of human skin fibroblasts BJ-5ta after 72 h of incubation with nanoparticles UCNPs–PEG and UCNPs–PSA.

The effective accumulation of UCNPs in a solid tumor depends on their circulation time in the bloodstream, which is directly associated with the adsorption of blood proteins. Nonspecific adsorption was assessed by the content of proteins in the supernatant (by the Bradford method) after centrifugation of the samples that were preincubated in a mouse blood serum solution (37°C, 1 h). The high protein concentration in the supernatant indicated an insignificant amount of protein adsorbed on the UCNP surface (Fig. 2a).

This allowed us to conclude that the PSA coating, in comparison with polyethylene glycol (PEG), which is recognized as the leader among modifier polymers for in vivo studies [9], largely prevents the formation of a “protein crown,” which can lead to the aggregation of UCNPs and their rapid withdrawal from the bloodstream.

When obtaining reagents for in vivo studies, it is also necessary that UCNPs are “invisible” to cells in the bloodstream (in particular, they do not induce phagocytic activity). The level of UCNP–PSA entrapment by cells in comparison with the PEG-modified UCNPs was assessed using RAW 264.7 mouse macrophages (Figs. 2b) by flow cytometry with the rhodamine B dye preliminarily incorporated into the nanocomplex. It should be noted that UCNP–PSA nanocomplexes accumulated in the cells to a lesser extent, that is, the level of their entrapment by phagocytes is lower.

Assessment of chronic toxicity of the UCNP–PSA nanocomplexes showed that, starting from a concentration of 100 mg/mL, an almost 100% survival of human skin fibroblasts BJ-5ta was observed after 72 h of incubation (Fig. 2c), which is comparable to the cytotoxic properties of the UCNP–PEG complexes.

**Fig. 3.**
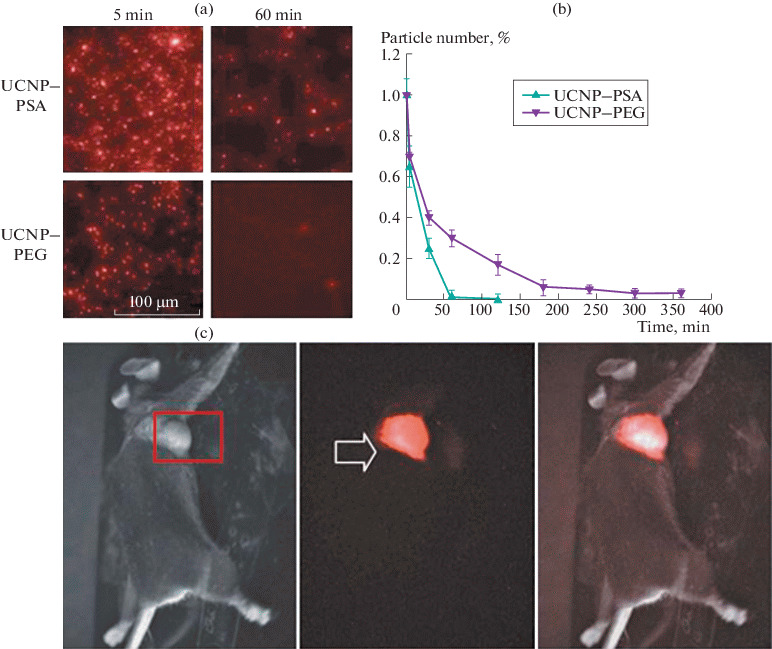
(a) Photoluminescent images of mouse blood samples taken at time intervals of 5 and 60 min after intravenous injection of UCNP–PEG and UCNP–PSA. (b) Dependence of the amount of UCNP–PEG and UCNP–PSA in the bloodstream on the nanoparticle circulation time. (c) In vivo images of Balb/c mice 1 h after injection of UCNP–PSA (brightfield image (left), fluorescent image (center), and superposition of images (right)).

An analysis of the circulation time of UCNP–PSA in comparison with UCNP–PEG after intravenous injection to Balb/c mice was performed by counting nanoparticles in blood samples (Figs. 3a, 3b). The data obtained allow us to conclude that the circulation time increased almost 3 times as compared to the PEG-modified UCNPs, for which the circulation time did not exceed 1 h.

The significant increase in the circulation time of nanoparticles in the bloodstream contributes to the efficient accumulation of nanoparticles in the solid tumor due to the EPR effect. The distribution of the signal from UCNP–PSA in mice with transplanted Lewis lung carcinoma after the injection of UCNP–PSA into the retroorbital sinus of mice was recorded using the epiluminescence imaging system developed at the Federal Research Center “Crystallography and Photonics,” Russian Academy of Sciences [10]. The photoluminescent signal appeared in the tumor 1 min after the injection, being slightly attenuated 2–4 min later due to partial withdrawal of the nanoparticles from the bloodstream and the redistribution of UCNP–PSA nanocomplexes in organs, and reached maximum 1 h after injection (Fig. 3c). The photoluminescence of UCNP–PSA nanocomplexes in the tumor was retained for 10 days.

## CONCLUSIONS

A method for biofunctionalization of UCNPs using polysialic acid was developed, which made it possible to obtain bioreagents characterized by low adsorption of blood proteins, low uptake by macrophages, and low cytotoxicity. The use of PSA for the biofunctionalization of UCNPs led to an increase in the circulation time of the obtained bioreagents in the bloodstream to 3 h, which underlies the efficient delivery and accumulation of UCNPs in the tumor due to the EPR effect. Thus, the UCNP–PSA nanocomplexes are very promising for use as bioimaging agents and as carriers for drug delivery. The proposed method for surface biofunctionalization using polysialic acid can be implemented for various nanoparticles, such as quantum dots, magnetic, plasmonic, etc., nanoparticles, for bioimaging and delivery of drugs and photosensitizers to tumors.
